# What Can We Learn from Police Data About Timeliness in Rape and Serious Sexual Offence Investigations in England and Wales?

**DOI:** 10.1007/s43576-022-00067-w

**Published:** 2022-10-13

**Authors:** Jo Lovett, Gavin Hales, Liz Kelly, Aneela Khan, Margaret Hardiman, Louise Trott

**Affiliations:** 1 London Metropolitan University, London, UK; 2 University of Suffolk, Ipswich, UK; 3 Bournemouth University, Poole, UK

**Keywords:** Rape, Sexual offences, Police, Investigations, Timeliness, Timescales, Crime data

## Abstract

The issue of timeliness in rape and other serious sexual offence investigations has been raised in a number of inspections and reviews, and there are policy imperatives to decrease delays, but there has been little exploration of police data to understand what contributes to them and enable practical recommendations or options. This paper explores what official data from two police forces participating in Operation Soteria Bluestone tell us about the timelines of these investigations, what this reveals about the gaps in policing data, and what additional knowledge can be gained from qualitative methodologies, in this instance case file analysis and case reviews.

## Introduction

The length of rape investigations has been raised as a concern for at least three decades, internationally and in England and Wales specifically, with various policy changes introduced to speed up processes (for example, early evidence reviews between police and the Crown Prosecution Service (CPS) (CPS & NPCC, 2021)) and ensure victims^1^ are kept updated in a timely manner (Victims’ Code of Practice) (MoJ, 2020). This is not just the case for investigations resulting in a trial; a recent inspection also found there to be an ‘unacceptable delay’ where there is a decision by police or CPS to take no further action (HMICFRS & HMCPSI, 2021, p. 46). The impact of Covid-19 on agencies and courts has added further layers of delay to a system that has been described as ‘broken’ (McManus & Almond, 2019), as increasing volumes of recorded rapes have been met with decreasing numbers of cases referred to the CPS for charge and prosecution, resulting in a falling conviction rate. The recent end-to-end rape review (HM Government, 2021a) called for ‘improved timeliness’ at all stages of the criminal justice system (CJS).

An ongoing research project with five police forces—Operation Soteria Bluestone^2^—was commissioned to explore what is and is not working in investigations of reported RASSO cases. Pillar 5 (one of five thematic areas the project comprises) focuses on data, in particular three years of quantitative data tracking aspects of the reporting and investigation timeline, and a smaller sub-sample of cases that were closed with outcomes 14, 15 and 16 (three Home Office crime outcome categories where there is no further action). Pillars 1 and 2 focus on suspects, and their work in each force has included facilitating officers to review investigations conducted by their peers. The main three-year dataset is limited for analysing timescales, since only certain dates are recorded in downloadable fields. The sub-sample deep dive of outcome 14, 15 and 16 case files adds to the knowledge base by offering additional detail of the timings of police actions and identifying the reasons for delay and drift, while the Pillars 1 and 2 case reviews provide further reflections on this from police officers. The smaller datasets derived from these qualitative reviews contain some material on the impact of timescales and delays on victims themselves through police representations of their perspective. A separate research pillar within the project focuses on the victim experience (Hohl et al., 2022, this issue), but there is not scope to draw on findings here.

This paper contributes to the evidence base on timeliness by showing the impact on this of suspect-victim relationship type, as well as highlighting the barriers within existing police data systems for both police and external researchers to monitor it effectively. We present key findings about the timing and length of RASSO investigations from two police forces and draw attention to a number of gaps in existing police data, some of which are addressed by the dataset produced by researchers during the review of outcome 14, 15 and 16 case files. By utilising additional methods, such as analysis of case files and officer-conducted case reviews, we are able to highlight some of the reasons for delays. The findings have implications for police recording practices and understanding of—and efforts to address—the timescales and factors associated with attrition in sexual offence cases.

Operation Soteria Bluestone was tasked with focusing on RASSO (rape and serious sexual offences), a term in general use within the policy and criminal justice arenas, but there is no current standard or consistent definition of exactly what offence types this consists of. The approach taken in this paper is that RASSO broadly encompasses rape and other penetrative and contact sexual offences.^3^

### Background Context

Recent data show that, in England and Wales, rape cases are among the lengthiest of any offence type, taking longer for the police to assign an outcome and, for the few that make it that far, in the number of days elapsing from charge to completion at court (ONS, 2020). It takes on average 73 days to assign an outcome to a sexual offence, compared to 15 days for a violence against the person offence, and six days for all other crimes (ONS, 2018). The overall mean from police referral to the CPS charging decision is 39 days, whereas for adult rape cases it was 122 (HM Government, 2021b). Between 2010 and 2019, the median time for rape prosecutions to progress from offence to completion doubled from around 400 days to 800 days (with average values roughly four times as long), while for all offences over the same period the median rose from around 130 to around 160 days (with averages around one fifth longer) (MoJ, 2019). Child rape cases take even longer to conclude: on average 1591 days longer than an adult offence (ONS, 2018).

In research conducted for the Victim’s Commissioner (Molina & Poppleton, 2020), 65% of rape victims surveyed about their experiences of the CJS had experienced ‘unreasonable’ delays in the investigation. While certain cases include a level of complexity that may require longer investigations (ONS, 2020), these extended timelines have a series of consequences for victim-survivors (Angiolini, 2015; Brooks-Hay et al., 2019; George & Ferguson, 2021; HM Inspectorate of Prosecution, 2017; Kelly et al., 2005), especially as the potential disclosability of therapy notes may lead victims to delay accessing therapeutic support until the legal case is concluded.^4^ They may withdraw their support from the criminal case because they lose trust in the process or simply decide that they cannot keep their lives on hold any longer. Victim withdrawal is significantly higher in rape cases, compared to other crime types (MOPAC, 2021). For example, victims of robbery, criminal damage and arson, and theft did not support police action in 17.7%, 16% and 7.5% of cases, respectively, whereas this was 41% for rape (Home Office, 2020). This difference is partly due to the much lower likelihood of identifying suspects in these types of reported crimes compared to rape.

The potential contribution of the length of investigations, intrusive nature of requests for mobile phones and third-party data (medical, social services, education and therapy records) and long periods where there is minimal case progression have been floated as reasons for victims’ withdrawal of support (ONS, 2018). All of these factors introduce delay into the process. Hohl and Stanko (2015) found that those who attended a Sexual Assault Referral Centre and, therefore, received support and medical care, were 44% less likely to withdraw support from the investigation, and the importance of support for victims was confirmed by the most recent London rape review (MOPAC, 2021). Furthermore, this study found that where victims completed a video recorded interview (VRI), they were 11 times less likely to withdraw from the case (MOPAC, 2021). While it is hard to untangle whether SARC attendance or undertaking a VRI is a function or a driver of victim support for the investigation, this suggests that completing these processes promptly is good practice, albeit some victims may want longer to decide. Dworkin and Schumacher (2018) also found that victims who are provided with community and psychological support at an early stage are less like to experience post-traumatic stress.

The backdrop to the research presented here is changing trends in the volumes of sexual offences recorded by the police, which have seen periods of both stability and sharp increases over the last four decades. The numbers of recorded sexual offences were relatively stable from 2004/5 to 2012/13 but recorded rape, for example, increased 3.6-fold in England and Wales between 2012/13 and 2019/20, from 16,374 to 58,845.^5^ In 2020/21, a decrease was recorded, undoubtedly linked to the impact of Covid-19 lockdowns on social life: rapes reported and recorded in the first quarter of 2020/21 were conspicuously low at 11,800, well below the quarterly average across 2019/20 and 2020/21 of 14,300. At the same time, the prevalence of sexual offences, as reported to the Crime Survey England and Wales, appears to have remained relatively unchanged. This suggests that the increase is more likely to have been driven by changing police crime recording practices (George & Ferguson, 2021), notably following the renewed scrutiny of crime recording after 2014 (Home of Commons PASC, 2014), including inspections undertaken by Her Majesty’s Inspectorate of Constabulary, Fire and Rescue Services (HMICFRS).^6^ There has also been an increase in reporting of non-recent sexual violence, connected to high-profile cases and improved understanding of sexual violence more generally (ONS, 2018).

This could lead to the suggestion that the length of investigations is simply a function of the substantially increased volume of cases coming to police attention. However, changing CPS requirements and the lengthy timescales involved in obtaining mobile phone downloads, analysis of forensic samples and third-party material are also likely contributors. Another factor in the length of investigation may be resources, with successive governments for more than decade presiding over falls in the numbers of police officers and support staff; by 2021 there was a shortfall of more than 6800 PIP2^7^-accredited investigators in the police service (NPCC, 2022, p. 65). This is understood as a combination of austerity measures and localism, contributing to a more fractured policing structure with less accountability, fewer officers and higher caseloads, with implications for expertise and the time available for each investigation (Solar & Smith, 2018; Topping, 2021). Mann et al. (2018, p.639) note that these processes have resulted in “a disregard for the importance of specialist knowledge and skills”, and in particular that “sex offender management units [are] being under-skilled and overwhelmed by demand”. HMIC (2016) viewed sex crimes as "for the most part a specialist area of policing” (p. 69), but there are widespread concerns that broader changes have reduced specialisation.

This context of increased volumes of offences and austerity has affected all parts of the CJS in two related ways.^8^ Firstly, CJS timescales have extended; as noted above, the median time elapsed for rape prosecutions to progress from offence to completion doubled between 2010 and 2019. Secondly, charge and prosecution rates for all offence types, but notably for rape, have fallen—although this is partly a function of when charge rates for a given year are calculated, as they continue to rise over subsequent years as more cases reach a conclusion. As at January 2022, the charge rate for rapes recorded in 2016/17 stood at 9.2%, compared to 2.2% for those recorded in 2020/21 (Home Office, 2022, and previous iterations).

Whilst the findings reported here can only speak to some of these issues, they nevertheless expand what we know about delay and drift in RASSO investigations.

## Method

This paper draws on selected data from two police force areas (referred to below as Force 1 and Force 2) that are among five forces participating in Operation Soteria Bluestone. The five forces were selected by the project funders to include large urban and smaller more dispersed force areas in England and Wales. The two forces from which these findings are drawn are included here because the data collection in these areas has been completed, while work in the remaining three forces was still ongoing at the time of writing. The two force areas are geographically diverse. Force 1 covers a large physical area combining urban and rural districts while Force 2 is a large metropolitan force. At the time of the research, both had moved away from specialist approaches to investigating RASSO and were operating an omnicompetence model, meaning that officers deal with a wide range of crime types. Compared with other forces, nationally, both were also at the lower end of the scale in terms of charge rates.

Operation Soteria Bluestone comprises five academic research teams^9^ using mixed methods to collect data across multiple strands. Here we draw on three data sources gathered by two of those teams that have particular relevance to the question of RASSO investigation timescales: quantitative police data on RASSO offences recorded from 2018 to 2020 (referred to as the ‘three-year dataset’); case files finalised as outcome 14, 15 and 16; and case reviews (see Table 1). These datasets all cover the investigation process and the timings of key stages within it. The three-year dataset provides the overall picture in each force in terms of the volume of reports, case profiles and outcomes. The case reviews and case file analysis are separate sub-samples drawn from the same period. They provide the opportunity for additional quantitative coding and analysis, as well as more in-depth qualitative analysis. The three datasets complement each other, providing a more complete picture of timeliness and enable discussion of some wider contextual factors affecting it.

**Table 1:** Summary of data sources used

Data strand	Dataset referred to as	Approach to sampling	Force 1 N	Force 2 N
3-year police force data	3-year dataset	All RASSO cases reported to force 2018–2020	10,625	36,921
Case file analysis of outcomes	Case file dataset	Most recently closed rape cases finalised as Outcome 14, 15 and 16 prior to the research in 2021	Not undertaken	294 *Outcome 14 – 98* *Outcome 15 – 96* *Outcome 16 – 100*
Case reviews	Case review dataset	RASSO cases sampled from 2019–2021 across three different outcome groups: victim declined to prosecute (outcome 14/16) no further action (outcome 15/18), and charged, and three suspect-victim relationship types (intimate, acquaintance and stranger)	38	50

Qualitative analysis of the case file and case review samples allows for a more nuanced understanding of timescales and provides specific examples that both pinpoint the causes of delays within investigations according to the officers themselves and highlight the impact these delays can have on case outcomes and on victims’ support for the investigation. These datasets offer specific insights not previously available: on officer, supervisor and crime management unit decisions and rationales for case disposal; factors contributing to delay; and some limited data on victims’ perspectives on this. In the case reviews, through the process of peer reviewing colleagues' case files, officers critically reflected on standard practices in their forces and offered potential explanations for delays that were grounded in their own experience.

### Three-Year Datasets

All police forces in England and Wales use computer systems to record incidents/crimes that are reported to them, or otherwise discovered. There is no standardised system in use nationally, so it was unsurprising that Force 1 and 2 employed different ones, but these systems typically include a mixture of structured and unstructured (free text) fields capturing a range of data about each incident and what actions police have taken. Some data are mandated by the Home Office and must be reported to them in an Annual Data Return. This includes crime classification and counts (for the purposes of crime counting) and outcomes, so these tend to be gathered in a relatively consistent way across forces and both are subject to quality assurance processes. To ensure the data received from Force 1 and 2 were as comparable as possible, the research team submitted a data request to them specifying inclusion criteria and a list of variables required. Some recoding was also conducted before analysis was undertaken.

Both Force 1 and 2 collected structured data on the date (or earliest date) on which the offence was alleged to have occurred, the date the allegation was reported to and recorded by police, and the date an outcome was assigned to a crime record. Force 1 also collected structured data on the date on which the victim was first contacted (and the number of contacts over time), and Force 2 on whether and when a suspect was arrested.

The three-year datasets from Forces 1 and 2 consist of all RASSO cases reported to police in the calendar years 2018 to 2020: 10,625 cases in Force 1 and 36,921 in Force 2.^10^ Whilst the two forces had slightly differing approaches to what constitutes RASSO, the data cover all recorded rape and other penetrative or contact sexual offences against victims of all genders and all ages. Anonymised datasets were produced by in force analysts, shared with the research team and then—following a number of queries, clarifications and iterations—subjected to exploratory and primarily descriptive quantitative analysis that sought to identify patterns and relationships in the data and between key variables. This included examining timescales associated with offence reporting and investigation, and how they varied by offence type, outcome and suspect/victim relationship.

### Case File Analysis

The case file analysis was only conducted in Force 2, building on initial work conducted in, and discussions with, Force 1. It concentrated solely on rape cases finalised according to three specific Home Office outcome codes: 14 (where a named suspect is not identified and the victim does not support further police action); 15 (where there is a named suspect identified and the victim supports police action, but evidential difficulties prevent further action); and 16 (where a named suspect identified but the victim does not support police action). It focuses, therefore, on cases that were not proceeding beyond the police stage. These three outcome codes accounted for 62% of rape allegation outcomes in the three-year dataset for Force 2 (respectively, 16.0%, 14.9% and 30.8%). The aim was to explore factors affecting discontinuance in these case types, especially where there was [reportedly] a lack of support for the investigation on the part of the victim.

To ensure the insights obtained related to current practice, 100 cases finalised under each outcome type were sampled from the period immediately preceding the research. Most of these were reported during the period of the three-year dataset, although a small number were more recent. A total of 294^11^ case files were examined (*n* = 98 outcome 14; *n* = 96 outcome 15; and *n* = 100 outcome 16). The case files consisted of a textual version of the electronic record on the force database, and the primary information they contained was a detailed narrative log documenting the investigation, with entries from each officer and other police staff involved. Excerpts from interviews and statements were sometimes included here. This content was extracted by Force 2 in PDF format and redacted before being shared securely with the research team. Cases were analysed to identify key dates and qualitatively explore factors affecting discontinuance. Quantitative data, such as victim, suspect and offence characteristics, were coded to enable descriptive analysis. The dates of key points in the investigation, including report, victim and suspect interview, and case closure were also plotted to explore investigative timescales. Qualitative data on evidential issues and rationales for case outcomes were subjected to thematic content analysis in order to generate descriptive codes about why cases were closed. To ensure extraction was consistent across the research team, quantifiable coded data were recorded in an Excel spreadsheet, while a descriptive summary of the investigation was recorded within a timeline in a qualitative pro forma. The application of the descriptive codes was discussed at a research team level to ensure consensus.

### Case Reviews

A total of 88 case reviews were conducted (*n* = 38 in Force 1 and *n* = 50 in Force 2). Cases were sampled across three different outcome types (victim does not support police action (outcome 14 and 16), no further action (outcome 15 and 18^12^) and charged) and three suspect-victim relationship types (intimate, acquaintance and stranger). The sampled cases underwent internal review, with serving police officers independent of the case invited to reflect on the strengths and areas for improvement throughout the investigation process. A further review of each case was then conducted by a senior officer. Both the investigative log and reviews were analysed in order to discover what contributed to delays in RASSO cases. Content analysis was used to identify common patterns emerging from the dataset across both forces, specifically difficulties towards achieving timely investigative milestones.

Both forces used the same tool to collect information on case reviews in order to ensure that comparable data were captured. Due to the qualitative nature of the dataset, no data were considered missing if cells had not been completed. Rather, it would highlight specific areas of the investigation, for example, CPS engagement, that had not been taken into consideration within the case. Through the use of headings that encompassed specific factors within the investigation process, direct comparisons were able to be made between Force 1 and Force 2, both broadly and specifically across incident types and outcomes.

### Limitations

Police data are generally subject to important limitations, including data gaps due to key fields such as sex, age and ethnicity not being completed, as well as errors and inconsistencies, for example between victim sex and crime classification. Crime recording systems that do not use unique identifiers for individuals can present challenges when identifying repeat offending or victimisation, including because name data are recorded inconsistently due to abbreviations, spelling mistakes and common names being erroneously linked/matched. There are also complexities linked to the multiple potential layers of many-to-many relationships within the data, as cases can involve multiple victims, suspects and/or offences. Other structured variables may also include multiple options, for example, alcohol, drug use and mental health, which may all be recorded as victim vulnerabilities, and offence *modus operandi*. These complexities can introduce high rates of duplication when complex records are extracted into rows in a spreadsheet for the purposes of strategic analysis. In addition, data extracted from live police systems are frozen at a point in time, while open cases continue to be investigated and resolved. All of these issues present handling difficulties for analysts and researchers and can lead to discrepancies with 'official' published crime counts.^13^

The data reported on here include a period of lockdown in response to the Covid-19 pandemic. There is evidence that the pandemic has had an influence on recorded crime volumes and outcomes, with some recent increases observed in time to charge and closure in rape cases (Flatley, 2021). Therefore, timescales in our samples relating to 2020 may have been partly affected by this. However, although some case files referred explicitly to the pandemic, it was clear that numerous systemic and other issues were also involved.

## Findings

### Three-Year Datasets

The data on recorded rape allegations have been summarised in Table 2, presenting both average and median timescales due to the highly skewed nature of the data (with very extended timescales in some cases), more so in the case of Force 2.

**Table 2:** Timescales of reporting and outcomes for rape allegations in the three-year dataset

Rapes		Force 1				Force 2^a^			
		2018	2019	2020	2018–2020	2018	2019	2020	2018–2020
Offence to report (days)	Avg	1257.4	1351.6	1247.9	1287.6	1585.3	1516.7	1492.1	1531.4
	Median	2	2	6	3	25	14	19	19
Report to first police contact with victim (days)	Avg	13.5	12.1	8.3	11.4	n/a			
	Median	1	2	1	2				
Report to suspect arrested (days)^b^	Avg	n/a				47.9	53.1	31.7	44.4
	Median					4	4	2	3
Report to outcome being assigned (days)	Avg	205.1	153.6	107.9	161.6	213.3	192.9	141.7	182.9
	Median	133	120	105	117	106	87.5	72	88
*Report to outcome (days)—less blanks and under investigation*	*Avg*	*204.8*	*152.5*	*108.4*	*161.7*	*210.2*	*172.8*	*109.9*	*167.8*
	*Median*	*133*	*120*	*107*	*117*	*122*	*94*	*63*	*92*
*Total (days)—less blanks and under investigation*	*Avg*	*1462*	*1504*	*1356*	*1449*	*1796*	*1690*	*1602*	*1699*
	*Median*	*135*	*122*	*113*	*120*	*147*	*108*	*82*	*111*

^a^In the case of Force 2, we were advised that the main outcome date recorded on the force crime recording system offered a misleading impression of the timescales due to a three-stage process needed to assign an outcome to a crime record: an initial outcome code applied by the OIC (Officer in Charge), which is then supervised by their Sergeant before the record is referred to a central crime management unit for quality assurance. We were told the latter stage can involve delays that are not related to the investigative timescale and point at which the victim(s) is informed of the outcome. To mitigate this, our force contact worked with colleagues to devise a means of extracting the date of the ‘last update by the OIC’ to the crime record

^b^Where the suspect was arrested, and where the arrest date was after the report date

The time from report to outcome is strikingly similar in both forces. Although it appears to be getting shorter over time, this will be because outcomes like charges that take much longer will not have been reached yet for the more recent cases. We can see this in the case of Force 2 (see Table 3), where we have detailed data on outcome timescales for the three-year dataset. Charges take around one year and five months on average, whereas no crime/cancelled crime decisions average only seven weeks.

**Table 3:**
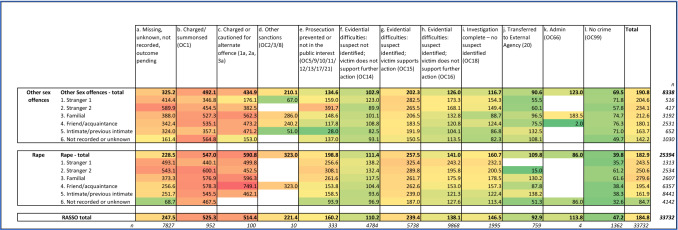
Average time elapsed between reported date and last entry on the file—a proxy for outcome date

The time to outcome varies somewhat by suspect-victim relationship. For example, for rape charges, the average time to charge after being reported to the police in stranger 2 cases is 600 days, while for stranger 1 it is 440.^14^ This may be related to the fact that, in contrast to stranger 2 cases, consent is unlikely to be a consideration in many stranger 1 rape allegations. With the exception of a single allegation, charges were only seen in stranger 1 cases where they were reported within the first week, namely within the forensic window.

**Fig. 1:**
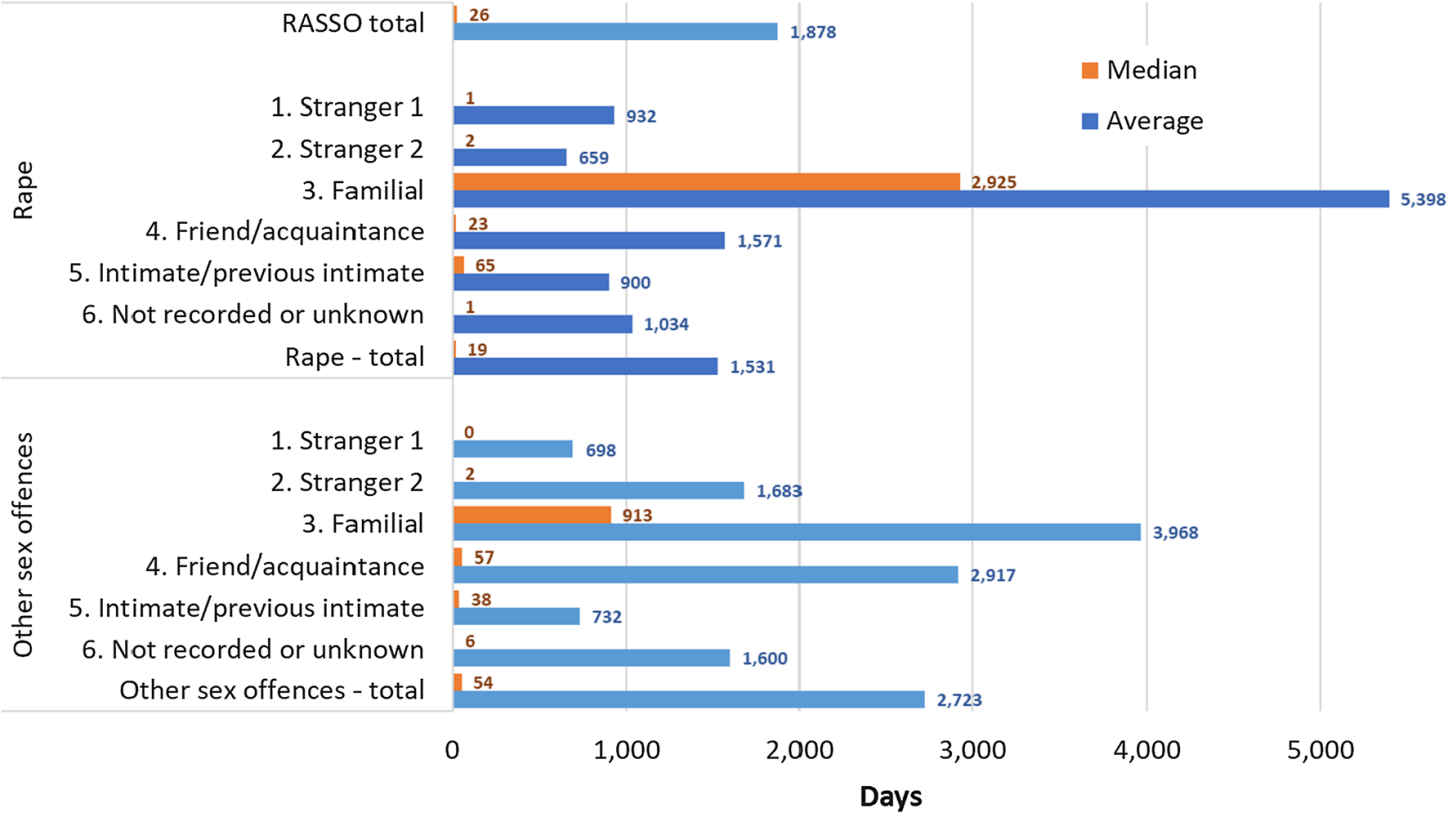
Average and median time from offence to report (days) by offence and suspect/victim relationship type (Force 2, excluding cases with no committed from date recorded)

There are also large differences in the time elapsed between the alleged offence and reporting to the police by the suspect/victim relationship: this was shortest in the case of stranger 2 rape allegations (average 659 days, median 2 days) and longest in the case of familial rape allegations (average 5398 days, median 2925 days) (Fig. 1 below).

**Fig. 2:**
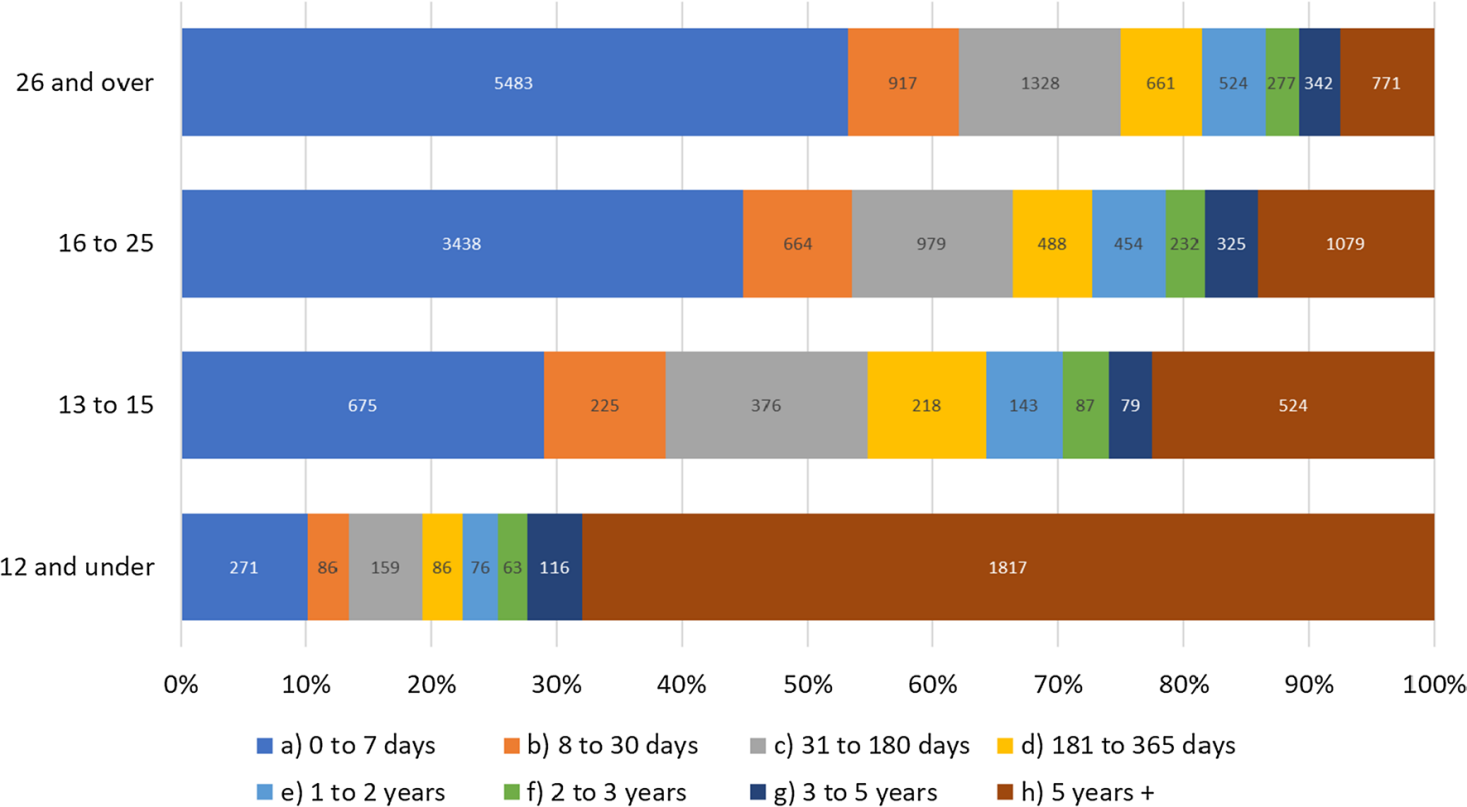
Rape victim age at time of offence and time between offence and report to police (Force 2, excluding victims with no age recorded)

Here it is significant that younger victims at the time of the alleged offences typically took longer to report to police, with 68% of reported rapes involving victims aged 12 and under at the time of the offence reported at least 5 years later, compared to only seven percent of reported victims aged 26 or over (Fig. 2 below).

These findings underline the importance of and need to control for relationship type in the analysis of all aspects of RASSO cases, as it appears to have a bearing on victim reporting, victim engagement, the salience of consent, and outcomes (especially the charge rate).

While structured data were available in both Force 1 and Force 2 for the dates (and therefore times elapsed) between alleged offences, reporting to police, reporting to suspect arrest (in the case of Force 2 only) and outcomes, most other key investigation process dates and milestones were not systematically recorded in a way that allowed them to be analysed, including with reference to investigation outcomes. This includes the dates on which statements were taken and forensic evidence submitted for (and results received back from) analysis, and dates relating to interaction with the CPS. This is not to say that the information is not available anywhere, but the use of free text case file updates to record such data means that systematic analysis would require individual files to be read and coded, which realistically is only possible for a small sample due to the time and effort required. In the case of Force 2, this was done through the case file analysis as part of our examination of case outcomes and attrition.

### Case File Analysis

The case file data enable more detailed analysis of the timeline, not only in relation to key actions in the police response, but also across the three Home Office outcome types, none of which proceed beyond investigation. As with the three-year dataset, there is wide variability in the data, so both average and median timescales are presented in Table 2. Where relevant, qualitative examples from the case files are provided as further illustration of how these delays play out in practice.

Within this dataset, the average length of time from offence to report date was around four years (median 5 days), which was at similar levels to the three-year dataset, but this was longest for outcome 14 cases (see Table 2). This shows the impact of historic cases on overall reporting timelines.

Where the outcome code suggests victims did not support or withdrew support for an investigation (outcomes 14 and 16), few victim VRIs were conducted, especially in outcome 14 cases (*n* = 5). As noted above, previous research has linked completion of a VRI with a lack of victim withdrawal; therefore, it is possible that victims in these cases were ambivalent about proceeding from the outset.^15^ Where victim VRIs were conducted, they were typically held more than one week after the initial report in outcome 14 cases (median 11 days), and much sooner after the initial report in outcome 16 cases (median 2 days).

In the case file dataset, in one case that was transferred from another force, there was a six-week delay due to lack of a suitable interview suite. In the meantime, it was stated in the file that no outstanding actions could be completed prior to receiving the VRI. Having eventually completed the VRI, the victim was asked to complete a follow-up VRI to provide a clearer allegation against the suspect. At this point, she declined to proceed:

I asked if she still wished to do this and she said no, she’s got better things in her life to do and doesn’t want to keep thinking about it […] there is no point continuing as the police will never find this person, she will never see him again, it happened last year and she’s got better things to do in her life than carry on with a police investigation (Force 2, Outcome 14, Case 38).

The majority of suspects (*n* = 78, 81%^16^) were interviewed in outcome 15 cases, and well over half (*n* = 58; 58%) in outcome 16 cases, but just five^17^ in outcome 14 cases (5%). The latter figure is expected to be low, as outcome 14 is intended for cases where no suspect is identified (and victims do not support investigation). The greatest time between the initial report and suspect interview elapsed in outcome 15 cases (median 21 days).

The average time between report and initial case closure in outcome 14 cases was one month, confirming that investigations here were short-lived, whereas in outcome 15 cases it was closer to a year, with outcome 16 cases falling in between (see Table 4).

**Table 4:** Timescales of reporting and investigative actions in the case file sample

Rapes							
Time between each point in days	Outcome 14	*N*	Outcome 15	*N*	Outcome 16	*N*	Total
Offence to report							
Avg	1595.2	*85*	938.1	*93*	859.1	*89*	1111.7
Median	7		13		3		5
Report to victim VRI							
Avg	390	*5*	54	*60*	25	*19*	67
Median	11		8		2		7
Report to suspect interview							
Avg	461	*4*	85	*71*	17	*56*	70
Median	7		21		1		4
Report to initial closure/investigation end							
Avg	56.9	*98*	292.8	*95*	117.8	*89*	154.4
Median	27.5		223		64		58.5
Initial closure to final outcome assigned							
Avg	95.4	*97*	201.9	*94*	125.2	*89*	141.6
Median	30		97.5		65		57
Report to final outcome assigned							
Avg	152.2	*97*	497.4	*94*	242.9	*89*	294.9
Median	68		339.5		172		152.5
Total^a^		***98***		***96***		***100***	

^a^Totals for each point in the timeline may differ from the overall totals per outcome group due to key dates not being available in the data

In order to explore the potential lag between the submission of cases for closure by supervisors and final completion by the crime management unit, we recorded both timings. The former can be seen as tantamount to the effective end of the investigation, while the latter is when the finalisation is administratively completed in accordance with Home Office counting rules. Our analysis shows a lag ranging from an average of 95 days in outcome 14 cases to 202 days in outcome 15 cases. If investigation lengths are calculated based on the date of final outcome allocation, they are likely to be inflated in forces where this end point of the timeline is extended. Table 4 shows that the time from report to final outcome allocation is, in fact, two to three times greater than that from report to initial closure. This is significant for victims, since they are informed that the case will not proceed at initial closure, so this aspect of timeliness—only visible in the case file dataset—suggests the picture is not as extensive as the final closure date timeline would suggest.

Examples in the case files indicated additional issues surrounding delays associated with accessing digital downloads and analysis of forensic samples, progressing cases with the CPS, and overall workloads. In one case involving a woman with learning disabilities, who disclosed being raped by her ex-partner in the context of reporting him for harassment, the victim agreed to hand over her phone for analysis, as she stated there was a message that contained an admission by the suspect of the rape. Delays occurred as the task of sifting through 250,000 items on her phone was shared across the investigating officer and colleagues, and there appeared to be no technical support to quicken this process. Police then had difficulty downloading the victim’s social media data in a readable format and asked her to download it for them. This process of obtaining and reviewing digital material took around one year, largely due to police having no technical expertise at their disposal. It took a further 14 months for the file to be prepared and submitted to CPS, by which time the victim was six months pregnant and anxious to reach an outcome. CPS ultimately advised NFA three years after the original report.

In another case, Early Investigative Advice was sought around two months after the initial report, resulting in an action plan for police to work through. Although the log stated that all of these actions were completed within three months and the file was ready to send to the CPS, it was a year before it was eventually submitted, during which time a comment was logged that the victim was “*frustrated with the length of time she [was] having to wait*”. It then took a further six months for CPS to return a decision of no further action (NFA).

A number of cases revealed significant early investigative activity followed by periods of lull and drift. One case beset with delays relating to digital data and CPS decision-making highlights a number of different pressures squeezing the investigating officer’s time, including a high caseload, leave periods and the allocation of a stranger offence perceived as high priority.

Currently [I] have a workfile [sic] of 36 crimes. I have had to deal with court enquiries for four court trials I have over the next 6 weeks. I have also been allocated two new cases to investigate this week, one of which has had to take priority, because it is a stranger attack in a public area. Will do asap. I am currently on a period of annual leave (Force 2, Outcome 15, Case 21).

In a separate case where a CPS action plan had been set, workload pressures hampered the investigating officer progressing with the case.

I have yet to progress any of the actions set by the CPS almost three months ago. This is very disappointing and due largely to a consistently heavy workload. I hope to be able to begin progressing some of these actions in the coming days/weeks (Force 2, Outcome 15, Case 52).

These data illustrate some examples of what more could be understood about the timeliness of specific actions in the investigative timeline if information within police case files were held in more systematised and structured formats.

### Case Reviews

Analysis of the investigative actions within the case reviews revealed several challenges that contributed to delays at different points in the investigation, some of which mirror those evident in the case files. The key issues included: (a) allocation of cases; (b) cross-force ownership of cases; (c) inconsistent and poor quality of supervision; (d) retrieval of information; and (e) victims not supporting the investigation.

#### Case Allocation

Delays in allocating a case to a specific OIC were evident across several cases. In one, for example, the victim received no contact for one month after the initial report, partly due to the lack of an assigned officer in charge (Force 1, Case 22). As a result, several investigative tasks that were not actioned until a year after the initial report meant the intelligence that was eventually gathered was rendered ineffective.

The absence of an OIC was detrimental to the investigation and led to significant delays (Force 1, Case 22 – Second Reviewer).

There were also challenges with case progression being delayed by officers in charge either going on leave or attending training. During this time, investigative parameters and actions were not set, decisions were not recorded, and the responsibility of the case was left to the officer investigating (Force 1, Case 18). In one case, an officer in charge went on leave 29 days after the initial report. During this period of leave (19 days), the victim was not contacted or updated regarding the case, with the victim’s ABE (Achieving Best Evidence) interview eventually taking place 40 days after the initial report (Force 1, Case 15).

#### Cross-force Investigation

Transferred crimes created delays while the division or force a case belonged to was identified and contacted, resulting in delays to victim engagement and investigative actions, including interviews with victims and suspects and intelligence gathering. For one case, the length of investigation totalled 447 days, with the first month dedicated to cross-force investigation and a further three months allocating an officer in charge (Force 1, Case 16).

Due to the victim being in a County there are delays due to the [Force] and County batting between divisions (Force 2, Case 01 – First Reviewer).

In another case, the first reviewer noted the *“case was significantly let down by the fact that there were two forces not agreeing a joint plan to investigate”* (Force 1, Case 15). The dispute regarding the ownership of the case contributed to an investigation length of almost two years as the lack of effective cross-force investigation delayed standard investigative actions, meaning potential lines of enquiry were missed and safeguarding and risk assessment not co-ordinated.

#### Supervision

Delay was also connected to the poor quality and inconsistency of reviews by senior officers. There were two layers here. The first involved irregular reviewing of cases, leaving investigations to drift. In several cases (Force 1), the investigation continued for over a year, yet only received a minimal number of reviews from supervisors, with some of the first reviews occurring months after the initial report.

Investigation runs for approx. 3 years and there is no footprint from a DCI (Force 1, Case 11 – Second Reviewer).

The second was supervisors undertaking the review process as a tick box or checklist exercise rather than providing tailored guidance to OICs on a case-by-case basis. These reviews were more regular, but consisted of minimal information with no set action plan. In these cases, senior officers failed to review all of the information at hand and left detectives to lead their own investigations with minimal guidance. On the other hand, however, in one case (Force 1, Case 14) where action plans were set by supervisors after review, the officer in charge of the investigation failed to carry out the task, resulting in a lack of progression within the case.

#### Retrieval of Information

Significant delays across many cases were a result of awaiting the results of forensic processes, including the retrieval of information from digital devices. In one case, it took approximately nine months to receive the results of a download from a victim’s phone (Force 1, Case 36). Other sources of delay included obtaining results from forensic evidence and toxicology submissions, and third-party information from other external organisations, such as medical records.

There is mention of elimination DNA not being sealed properly meaning it could not be used so another sample was needed… this did cause some delay (Force 2, Case 01 – Second Reviewer).

In some cases, minor mistakes had major impacts, for example, the incorrect labelling of a forensic exhibit in one case led to a 45-day delay (Force 1, Case 02).

According to many reviewing officers, awaiting information from CPS was a significant source of delays. For example, in one case there was no response from CPS for three months due to a backlog of cases (Force 1, Case 06) and in another, there was a delay of four months between the submission of the case for a charging decision from CPS, allowing the suspect to flee the country in the meantime (Force 1, Case 02).

#### Victims not Supporting the Investigation

Throughout the first and second reviews, officers often stated that delays were a result of the “*victim not being willing initially*” (Force 2, Case 15—First Reviewer), suggesting that the length of the investigation was somewhat dependent upon the cooperation of the victim. This was further reiterated when one officer noted that “*once the victim engaged, the case progressed well*” (Force 2, Case 41—First Reviewer).

Of the 88 cases, the victim did not support the investigation in less than a quarter (23%). No cases where victims were not supportive in Force 2 (*n* = 13 cases) appeared to be a direct result of delays in the case. However, where victims were not supportive in Force 1 (*n* = 9), all of the themes outlined above were present within the investigations. In one case, the officer states that *“the delays in this case (it took well over a year to complete even the limited actions) had a huge bearing on this support for the investigation”* (Force 1, Case 22—First Reviewer).

## Reflections

This analysis of both three-year quantitative data and qualitative case file and case review datasets shows that while timeliness figures are often presented in global terms for all rape or RASSO investigations, this masks the variation in timescales for different outcome types and case profiles, particularly suspect-victim relationship. There are important differences in the timescales for outcome finalisation, and whether and how key actions and dates are recorded within police systems in structured fields. Both have serious implications for interrogating whether the timing of these actions is in line with policy and force guidance, let alone enhancing understanding of delay and drift and how these might be addressed. Currently, it is simply not possible to track timeliness in completing the basic components of an investigation at the strategic level because too many dates are buried in free-text investigation logs.

Delays in finalisation mean that officers are still technically carrying cases they are no longer investigating, although these may require certain administrative actions to be completed before they can be deemed closed. This has implications for officer morale and may impinge on their capacity to deal with live investigations. It is important that there are processes in place to prevent cases being closed inappropriately, but resources are required to ensure there is adequate capacity to do this in a timely fashion.

Crime management processes also have a bearing on how investigative timescales can be calculated, raising the question of which point we should take as indicative of when a case is actually closed—for example, when the case is submitted for closure, or when closure is confirmed by the crime management team? The period between the two can be particularly protracted where there are disagreements about which outcome code to apply or where further administrative actions are required to close according to protocol, although some cases were closed relatively quickly, such as outcome 14 cases.

Case file analysis across three outcome types that do not proceed to charge, and of the additional timeliness data that access to the case files afforded, showed substantial variations between the three in reporting and investigation timescales, as well as in whether and when victim and suspect interviews were conducted.

Longer investigations have implications for victims, who may be deterred by the time taken to conduct basic police processes and find it impossible to maintain their support of the process over months, if not years. Examples of delay and drift were evident in both the case files and case review data, and they provided some detail on factors affecting these delays, such as difficulties obtaining evidential material, ineffective supervision and workload.

Further research within Operation Soteria Bluestone will look at the timings of requests for third-party material and CPS early advice within the case files, as well as integrating findings that speak to victims’ experiences of these delays. Through the case file analysis, we are also exploring at what stage victims’ lack of support for investigations is expressed within cases that are not proceeding.

## Conclusion

The end-to-end rape review has called for “improved timeliness of cases at each stage of the criminal justice process” and one of its proposed actions is the “consistent collection of data on timescales and progression of cases” in order to “hold each element of the criminal justice process to account” (HM Government, 2021a, p. 16). For this to be the case, management systems and supervision need to ensure that key date fields are present and completed so that performance monitoring can routinely track the timing of key investigative actions and attrition points in order to assess where inefficiencies lie and where practice could be improved, and this should be reported on in force wide strategic and problem profiles. This paper has shown that the capacity to fill in some of the data gaps offers opportunities to identify where improvements could take place if timeliness became a focus for improvement. Only when data are easily retrievable and police forces give timeliness the attention it deserves will accountability and improvement be possible.

## Footnotes

1 We are alive to the debates and significance of language in relation to the terms ‘victim’, ‘survivor’, ‘victim-survivor’ and ‘complainant’. We use ‘victim’ here, primarily, in recognition of the fact that we are discussing individuals within the criminal justice process and in the context of how they are recorded in police data.

2 Operation Soteria Bluestone is a UK Home Office-funded programme designed to improve the investigation of rape and serious sexual offences (RASSO) in England and Wales. It is a unique project which is underpinned by rigorous social science. With multi-disciplined academics located in multiple universities, mixed qualitative and quantitative methods are applied to a five pillared approach to organisational change with police forces, uplifting the capability of more specialist police decision-making in RASSO cases. The research informs policing practice as well as government policy and is set to inform a national change. These research informed pillars pinpoint specific areas for improvement which will form part of the new framework for investigating RASSO: (1) suspect-focused investigations; (2) disrupting repeat offenders; (3) victim engagement as procedural justice; (4) promoting better learning, development, and wellbeing for police officers; and (5) using data more effectively in RASSO investigations. The pathfinder project started in 2021, based in Avon and Somerset Constabulary. Designed by Katrin Hohl and Betsy Stanko, the pillar leads include Kari Davies, Miranda Horvath, Kelly Johnson, Jo Lovett, Olivia Smith, and Emma Williams.

3 While there may be minor differences between the two forces in which offences they have included as ‘RASSO’, these make a negligible difference to the results.

4 See https://rapecrisis.org.uk/news/new-crown-prosecution-service-guidance-will-block-rape-victims-from-therapy/

5 Between 2012/13 and 2020/21, the proportion of police recorded sexual offence allegations that were rape increased from 31 to 38% (Home Office, 2022).

6 See https://www.justiceinspectorates.gov.uk/hmicfrs/our-work/article/crime-data-integrity/reports-rolling-programme-crime-data-integrity/.

7 Professionalising Investigation Programme, a development programme for investigators. Level 2 focuses on serious complex investigations.

8 Between 2010/11 and 2018/19, total funding to police forces in England and Wales fell 19% in real terms (NAO, 2018).

9 See description of the five pillars in note 2 above.

10 Note that the exact totals vary depending on a number of decisions about data handling and cleaning during analysis.

11 Out of 300 requested files, six were discarded due to sampling error or file inaccessibility.

12 Outcome 18 is defined as ‘Investigation complete—no suspect identified’.

13 As an extreme example, one incident in Force 1 involved one victim and 10 suspects, as well as eight vehicles, with the result that the single incident resulted in 80 rows of data. More prosaically, crime records may, for example, include more suspects than perpetrators—such as where someone is assaulted at a party by two people, but is unsure which two they were out of the 10 present—so suspect numbers and crime counts may not reconcile.

14 In stranger 2 cases, there was some contact between victim and suspect before the alleged offence—for example, because they met in a social setting that evening. In stranger 1 cases, by contrast, there was no prior contact.

15 We are conducting further analysis of how outcome 14 and 16 cases are reported and whether or not victims initially sought a police investigation.

16 Timings data were only available in 71 cases.

17 Timings data were only available in four cases.
